# Scorpion venom peptide SPVII promotes irradiated cells proliferation and increases the expression of the IL-3 receptor

**DOI:** 10.1186/2045-3701-3-28

**Published:** 2013-07-08

**Authors:** Yifang Qiu, Liyuan Jiang, Caixia Wang, Yan Wang, Ting Li, Baiqian Xing, Meixun Zhou, Tianhan Kong, Weihua Dong

**Affiliations:** 1 Department of Pathophysiology, Guangzhou Medical College, Guangzhou 510182, Guangdong, China; 2 Department of B2B Human Disease, Beijing Genomic Institute (BGI), Shenzhen 518083, China; 3 Department of Hematology, Guangzhou First Municipal People's Hospital, Guangzhou 510182, Guangdong, China; 4 Department of Pathology, Jiangmen University of Chinese Medicine, Jiangmen, 529000, Guangdong, China

**Keywords:** Scorpion venom, Polypeptide, Radiation, Hematopoietic cells, Receptor, IL-3

## Abstract

**Background:**

The previous investigation demonstrated the radioprotective efficacy of peptides isolated from the venom of *Buthus Martti Karsch*. In this study, the effect of isolated scorpion venom peptide II (SVPII) on irradiated M-NFS-60 cells and mouse bone marrow mononuclear cells (BM-MNCs) was observed. The AlamarBlue cell viability assay, a colony-forming unit (CFU) assay, flow cytometry (FCM), immunofluorescence, and Western blotting were used to evaluate cell proliferation, cell cycle progression, and the expression of the IL-3 receptor (IL-3R) protein in non-irradiated and irradiated cells.

**Results:**

Proliferation of irradiated M-NFS-60 cells was significantly accelerated by SPVII, and this effect was further enhanced by co-application of IL-3. Similarly, SPVII increased the number of BM-MNC CFUs and this proliferative effect was greater in the presence of SVPII plus IL-3. In addition, SPVII significantly altered cell cycle progression; SVPII enhanced the fraction of unirradiated M-NFS-60 cells in S phase and the fraction of irradiated M-NFS-60 cells arrested in G2/M. The expression of IL-3R protein by unirradiated M-NFS-60 cells was enhanced significantly by SVPII, and SVPII-induced IL-3R overexpression was 10-fold greater in irradiated M-NFS-60 cells.

**Conclusions:**

These results indicated the hematopoietic growth factor (HGF)-like effects of SVPII on irradiated cells, possibly mediated by upregulation of IL-3R.

## Background

Irradiation therapy serves as one of the main treatments for malignant carcinoma. Radiotherapy kills cancer cells, but also injures actively proliferating healthy cells. Bone marrow is among the most vulnerable tissues to radiotherapy-induced damage. Irradiation may lead to hematopoietic failure, significantly decreasing the efficacy of cancer treatment and negatively impacting patient quality of life. The recovery of hematopoiesis relies on the proliferation and differentiation of undamaged hematopoietic stem cells (HSCs) under the regulation of a specific group of cytokines. Hence, recombinant cytokine treatment is the traditional therapy for mitigating the inhibitory effect of irradiation on hematopoiesis [1]. The most common drugs used to reverse hematopoietic suppression are colony stimulating factors (CFSs), including granulocyte CSF (G-CSF), granulocyte-macrophage CSF (GM-CSF) [2], and monocyte-macrophage CSF (M-CSF). However, the efficacy of these CSFs is limited and cytokine treatment also causes additional adverse events [3,4].

Agents that confer radiation-resistance have been studied for over 40 years. Thousands of potential agents have been investigated, including sulfur compounds and vitamins [5], plant-derived drugs [6,7] and cytokines [8]. However, most of these agents are unable to satisfy the requirements of effectiveness, low toxicity and specificity. Our previous research indicated that scorpion venom peptides (SVPs) protected against radiation-induced bone marrow injury, accelerated the formation of hematopoietic cell colonies following irradiation, and increased the levels of several cytokines in bone marrow and blood, resulting in enhanced recovery of hematopoiesis in irradiated mice [9-11]. Based on the outcomes of our preliminary investigation, the proliferation accelerating effect and mechanisms of SVPs on the cytokine-dependent M-NFS-60 cell line (ATCC: CRL-1838™), un-irradiated or irradiated, and primary mouse bone marrow mononuclear cells (BM-MNCs) were observed.

The proliferation of M-NFS-60 cells depends on both M-CSF and IL-3. Under cytokine treatment, M-NFS-60 cells rapidly proliferate but maintain the characteristics of immature bone marrow cells [12]. Therefore, M-NFS-60 cells are commonly used for studies on hematopoiesis. IL-3 promotes pleuripotent hematopoiesis by stimulating the self-renewal of early pleuripotent stem cells and the proliferation and differentiation of marrow-derived progenitor cells, resulting in the continued production and survival of mature blood cells [13,14]. Previous studies confirmed that IL-3 can protect bone marrow cells against radiation-induced apoptosis and regulate the expression of certain oncogenes such as c-myc. In addition, IL-3 protects bone marrow cells against DNA damaging agents [15]. In this study, M-NFS-60 and BM-MNCs cells were treated with either SVPII alone or in combination with IL-3. SVPII promoted the proliferation of irradiated M-NFS-60 cells and stimulated the colony formation of non-irradiated bone marrow cells. These effects were further increased when SVPII was combined with IL-3. Furthermore, SVPII significantly altered M-NFS-60 cells cycle progression, increasing the fraction of unirradiated cells in S phase and irradiated cells in G2/M. In addition, SVPII upregulated the expression of the IL-3 receptor (IL-3R), especially following irradiation, suggesting that the proliferation accelerating effect of SVPII on irradiated cells depends on activation of IL-3R-mediated signaling pathways.

## Results

### Effect of SVP on the proliferation of irradiation or non-irradiation M-NFS-60 cells

**Figure 1:**
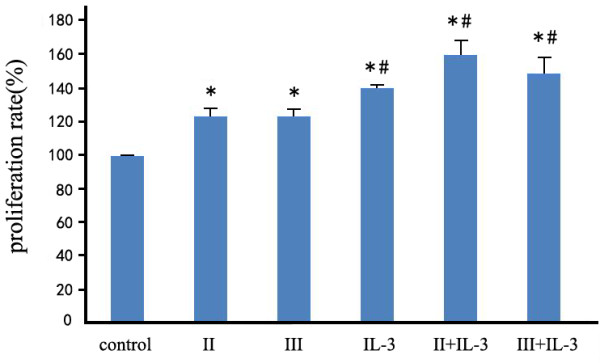
**SVPII and SVPIII enhanced proliferation of irradiated M-NFS-60 cells (applied alone or with IL-3).** M-NFS-60 cells were irradiated for 5 min (5 Gy total dose) and then treated with PBS (control), 3 mg/L SVPII for 48 h (II), 3 mg/ml SVPIII for 48 h (III), 10 μg/L IL-3 for 48 h (IL-3), or the indicated combinations (II + IL-3, III + IL-3). *P < 0.01 vs. control; #*P <* 0.05 vs. II or vs. III).

The proliferation of non-irradiated M-NFS-60 cells was markedly enhanced by treatment with scorpion venom proteins SVPII and SVPIII (1–3 mg/L for 24 or 48 h). Proliferation was greater at 3 mg/L than at 4 mg/L (data not show), so all subsequent experiments were conducted using the optimal concentration range of 1–3 mg/L. The proliferation of irradiated M-NFS-60 cells was accelerated by SVPII and SVPIII (3 mg/L for 48 h) as revealed by the AlamarBlue cell viability assay (*P* < 0.01; Figure 1). Proliferation was also enhanced by IL-3 alone (10 μg/L for 48 h). The combination of SVP (SVPII or SVPIII at 3 mg/L) plus IL-3 for 48 h exerted the greatest effect on cell proliferation (*P* < 0.01 vs. control; *P* < 0.05 vs. SVP alone; Figure 1).

Thus, both SVPs and IL-3 promoted the proliferation of irradiated M-NFS-60 cells and the effect of combined SVP + IL-3 treatment was more obvious. As SVPII + IL-3 exerted a larger proliferative effect than SVPIII + IL-3, SVPII was used in all the subsequent experiments.

### Effect of SVP on mouse hematopoietic cell CFU count

**Figure 2:**
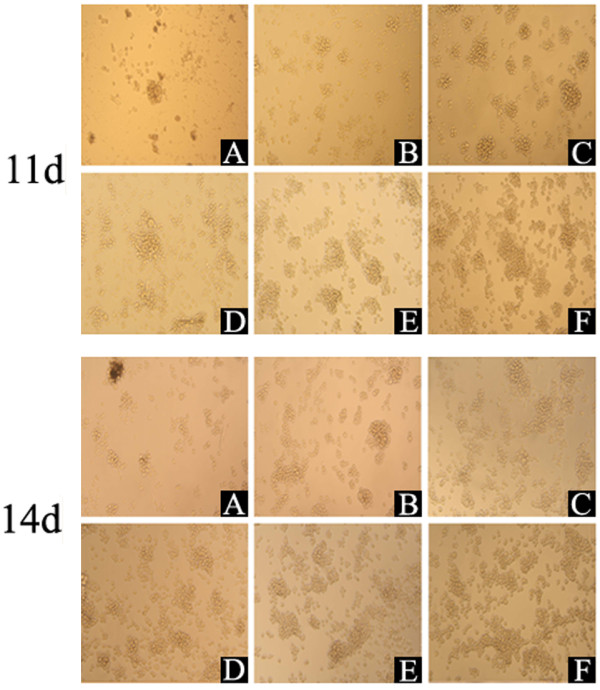
**Effect of SVPII on CFU count on 11th and 14th days in vitro (magnification: ×100).** BM-MNCs (1 × 10^6^ cells/mL) of BALB/C mouse were treated by: A: PBS (Control), B: cytokines (10 μg/L IL-3 and 62 μg/L rhM-CSF), C: 1 mg/L SVPII, D: 3 mg/L SVPII, E: 1 mg/L SVPII + cytokines, F: 3 mg/L SVPII + cytokines. Treated BM-MNCs were added into methyl cellulose half-solid medium and CFU was count on the 11th and 14th days.

BM-MNCs were isolated from BALB/C mice and used to examine the effect of SVPII on primary hematopoietic cell proliferation and survival. Isolated BM-MNCs were cultured for up to 14 days in methyl cellulose medium with SVPII (1 mg/L or 3 mg/L) or SVPll plus the cytokines IL-3 (10 μg/L) and rhM-CSF (62 μg/L). Treatment with SVPII alone increased the CFU count; the CFU count in 1 mg/L SVPII alone peaked on the 7^th^ day after administration and then declined (*P* < 0.05 on day 7 vs. controls), while the CFU count in 3 mg/L SVPII was higher on the 11^th^ and 14^th^ day compared to the 7^th^ day and significantly greater than PBS-treated controls on all measurement days (*P <* 0.05) (Table 1, Figure 2). The CFU number in cytokine-treated groups peaked on day 7 and remained significantly higher than controls on all subsequent days (*P <* 0.05). At all measured time points, the CFUs were higher in the 1 mg/L SVPII + cytokines group and the 3 mg/L SVPII + cytokine group compared to all other treatment groups, consistent with the synergistic effect of SPVII plus cytokines observed in M-NFS-60 cells. The CFU count in the 1 mg/L SVPII + cytokines group peaked on the 7^th^ day (*P <* 0.05) and then declined, while the CFU count in the 3 mg/L SVPII + cytokines group was higher on the 11^th^ and 14^th^ day compared to day 7 and significantly higher than all other groups on day 14 (*P <* 0.05).

**Table 1:** The effect of SVPII on the CFU of BALB/C mouse BM-MNCs (x̅ ± s)

**Groups**	**7d**	**11d**	**14d**
Control	69.50 ± 11.50	51.23 ± 8.76	29.00 ± 4.56
Cytokines	225.47 ± 14.24*	107.50 ± 22.74*	87.23 ± 16.38*
SVPII 1mg	197.50 ± 11.50*	62.50 ± 6.02	46.58 ± 6.78
SVPII 3mg	151.00 ± 26.35*	157.00 ± 27.39*^#‡^	184.00 ± 13.14*^#‡^
SVPII 1 mg + Cytokines	424.5 ± 32.74*^#‡△^	231.00 ± 18.62*^#‡△^	214.00 ± 15.37*^#‡^
SVPII 3 mg + Cytokines	315.50 ± 23.33*^#‡△^	286.50 ± 21.36*^#△‡^	305.47 ± 13.69*^#‡△^

*P < 0.05 vs. control; ^#^*P <* 0.05 vs. cytokine treated; ^‡^*P <* 0.05 vs. 1 mg/L SVPII; ^△^*P*<0.05 vs. 3 mg/L SVPII.

### Effect of SVP on the cell cycle progression of M-NFS-60 cells

As shown in Tables 2 and 3, SVPII promoted the proliferation of M-NFS-60 cells under serum-free conditions as evidenced by an increase in the total number of cells in S phase (*P <* 0.05 vs. serum-free controls) after both 24 h and 96 h treatment. In fact, the fraction of cells in S phase was significantly higher in M-NFS-60 cultures treated for 96 h with SVPII than in cultures treated for 96 h with IL-3 (Table 3).

**Table 2:** Effect of SVPII on M-NFS-60 cell cycle progression (24 h; % of total cells)

**Group**	**G0-G1**	**G2-M**	**S**
24 h Control	40.02 ± 5.25*	2.68 ± 4.58	56.76 ± 1.09*
Serum-free control	57.17 ± 1.66	2.67 ± 3.73	39.88 ± 1.95
SVPII 3 mg/L	39.92 ± 2.15*	2.17 ± 3.45	57.98 ± 5.68*
IL-3 10 μg/L	45.41 ± 3.63*	1.14 ± 1.67	53.13 ± 5.01*

The 24 h control cells were incubated in media containing 10% FCS + 62 μg/L rhM-CSF. All other cultures were treated with media plus rhM-CSF but without serum (serum-free media) or incubated in this same media supplemented with SVPII or IL-3 for 24 h. * *P <* 0.05 vs. serum-free control.

**Table 3:** Effect of SVPII on M-NFS-60 cell cycle progression (96 h; % of total cells)

**Group**	**G0-G1**	**G2-M**	**S**
96 h Control	45.51 ± 13.52	8.51 ± 2.95	41.97 ± 16.47
Serum-free control	71.56 ± 5.60	8.16 ± 5.10	20.29 ± 10.71
SVPII 3 mg/L	41.22 ± 14.96 *	4.29 ± 2.70	54.47 ± 17.66 *
IL-3 10 μg/L	51.81 ± 18.23	3.11 ± 1.40	44.88 ± 19.87

Cells were incubated in media containing 10% FCS + 62 μg/L rhM-CSF for 96 h (96 h control). All other cultures were treated with media plus rhM-CSF but without serum for 24 h and then treated for an additional 72 h with media supplemented with 10% FCS + 62 μg/L rhM-CSF (serum-free control) or treated with SVPII 3 mg/L or IL-3 10 μg/L in the same media. * *P <* 0.05 vs. serum-free control.

After irradiation by ^60^Coγ-ray, M-NFS-60 cells were incubated in culture medium containing 10% FCS, 15.5 μg/L rhM-CSF (25% of the normal dose), and 3 mg/L SVPII for 48 h and cell cycle progression compared to unirradiated cells, irradiated cells without SPVII, and irradiated cells treated with 10 μg/L IL-3 (Table 4).

**Table 4:** Effect of SVPII on irradiated M-NFS-60 cell cycle progression (% of total cells)

**Group**	**G0-G1**	**G2-M**	**S**	**Apoptosis**
Control	40.16 ± 3.40	6.69 ± 0.70	52.86 ± 4.83	0.01 ± 0.00
Irradiated	34.45 ± 1.75	31.71 ± 3.16	32.21 ± 2.65	15.81 ± 1.26
IL-310 μg/L	14.72 ± 0.91*	65.38 ± 5.11*	20.93 ± 2.76*	31.95 ± 4.11*
SVPII 3 mg/L	36.65 ± 2.07^#^	46.27 ± 4.28*^#^	16.92 ± 1.95*^#^	21.09 ± 2.05*^#^

Cells were unirradiated (Control), irradiated and then incubated for 48 h in media with 25% rhM-CSF (15.5 μg/L) for 48 h(Irradiated), or irradiated and then treated with 15.5 μg/L rhM-CSF + IL-3(IL-310 μg/L) or 15.5 μg/L rhM-CSF + SVPII(SVPII 3 mg/L). * *P <* 0.05 vs. irradiated group; ^#^*P <* 0.05 vs. IL-310 μg/L group.

After irradiation and 48 h incubation in media with 25% rhM-CSF (15.5 μg/L), 32.21% of M-NFS-60 cells were in S phase and 31.71% were in G2-M phase. For irradiated cells treated with IL-3 for 48 h, the proportion of cells in G2-M phase was significantly higher (65.38%), as were the percentage of apoptotic cells (31.95%). For the irradiated cells treated with SVPII for 48 h, 46.27% were arrested at G2-M phase, significantly higher than in irradiated group (drug-free). However, the percentage of cells in S phase was significantly decreased and the fraction of apoptotic cells was lower than in the IL-3 treatment group.

### Effect of SVP on the expression of IL-3R

#### Effect of SVP on the expression of IL-3R in M-NFS-60 cells

**Figure 3:**
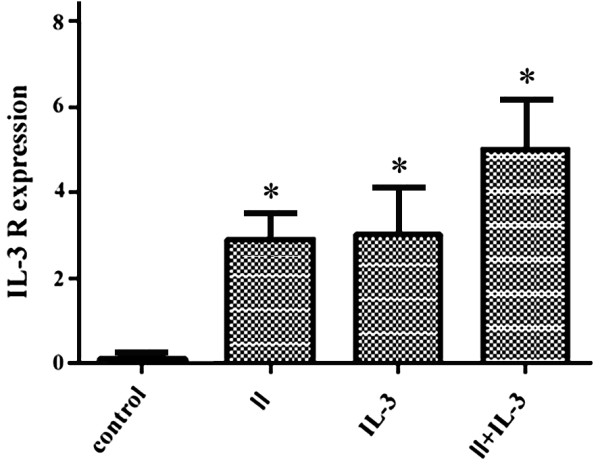
**Influence of SVPII on IL-3 receptor expression in M-NFS-60 cells (% IL-3R + cells).** Control: cells treated with solvent (PBS); II: cells treated with 3 mg/L SVP II; IL-3: cells treated with 10 μg/L IL-3; II + IL-3: cells treated with 3 mg/L SVP II and 10 μg/L IL-3 simultaneously. *P < 0.05 vs. control group.

**Figure 4:**
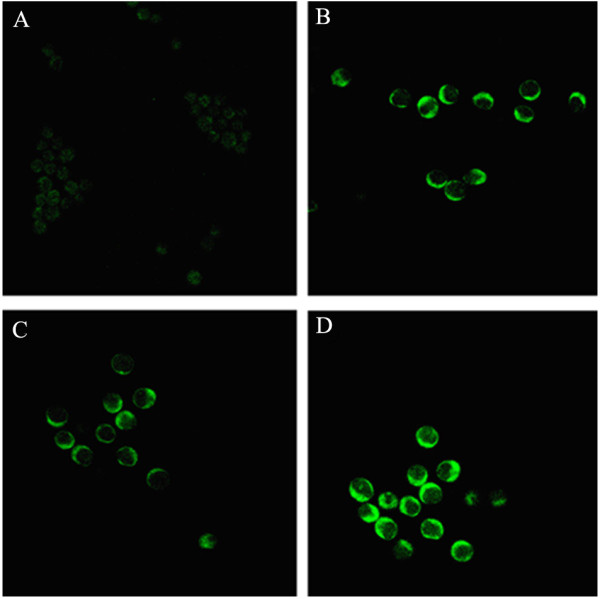
**Immunofluorescence images demonstrating the influence of SVPII on IL-3 R expression by M-NFS-60 cells (×200). A.** Control: cells treated with solvent (PBS); **B.** IL-3: cells treated with 10 μg/L IL-3; **C.** SVPII: cells treated with 3 mg/L SVP II; **D.** SVPII+IL-3: cells treated with 3 mg/L SVP II and 10 μg/L IL-3 simultaneously.

Following 48 h SVPII treatment, the expression level of IL-3R in M-NFS-60 cells was detected by FCM and cell immunoflurorescence. Flow cytometry indicated that the expression of IL-3R was upregulated after SVPII treatment and further enahanced by SVPII plus IL-3 (Figure 3). Immunofluorescence yielded similar results (Figure 4). The highest fluorescence intensity was observed in the SVPII + IL-3 group, followed by the IL-3 group, SVPII group, and normal controls (lowest), suggesting that the enhancement of M-NFS-60 cell proliferation by SVP may be associated with upregulation of IL-3R.

**Figure 5:**
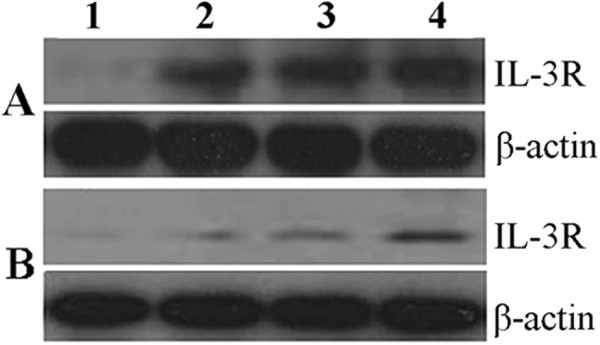
**Influence of SVPII on IL-3R expression by M-NFS-60 cells. A.** Normal culture condition: 10%FCM + rhM-CSF 62 μg/L); **B.** Low concentration of M-CSF: 10%FCM + rhM-CSF 15.5 μg/L; 1. Control: cells treated with solvent(PBS); 2. IL-3: cells treated with 10 μg/L IL-3; 3.SVPII: cells treated with 3 mg/L SVP II; 4. SVPII + IL-3: cells treated with 3 mg/L SVP II and 10 μg/L IL-3 simultaneously.

The growth of M-NFS-60 cells depends on the cytokine M-CSF. As the expression of IL-3R will be induced by M-CSF, IL-3R expression in response to IL-3 or SVPII was measured at normal M-CSF dose (62.5 μg/L) and 25% of the normal M-CSF dose (15.5 μg/L). Western blotting results revealed that SVPII significantly upregulated the expression of IL-3R at both M-CSF doses, while SPVII plus IL-3 exhibited a strengthening effect on IL-3R expression (Figure 5).

#### Effect of SVP on the expression of IL-3R in irradiated M-NFS-60 cells

**Figure 6:**
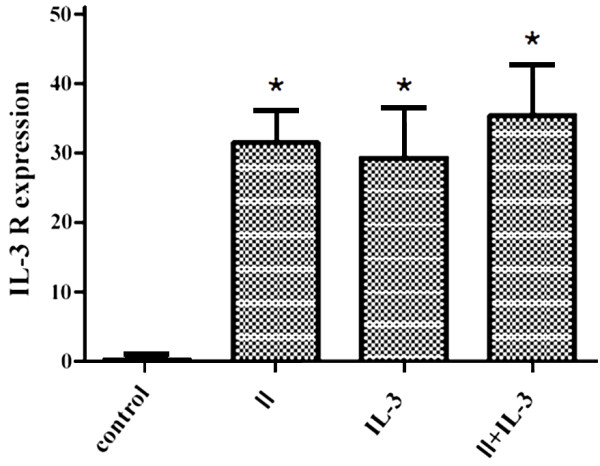
**Influence of SVPII on IL-3 R expression of M-NFS-60 cells after irradiation (% IL-3R + cells).** After irradiated, the cells were treated with solvent(control) or 3 mg/L SVP II(II) or 10 μg/L IL-3(IL-3) or with 3 mg/L SVP II and 10 μg/L IL-3 simultaneously(II + IL-3). *P < 0.05 vs. control.

Westerm blot and immunofluorescence results strongly suggested an association between the proliferation-promoting effect of SVPII (and SVPII plus IL-3) and upregulated expression of IL-3R, at least in unirradiated M-NFS-60 cells. In irradiated M-NFS-60 cells, the expression level of IL-3R was also significantly upregulated by 48 h of SVPII treatment and further enhanced by combining SVPII and IL-3 (Figure 6). Indeed, expression was approximately 10-fold higher than in SVPII- or SVPII + IL-3-treated unirradiated cells, underscoring the possible role of IL-3R overexpression in SVPII-mediated hematopoietic cell proliferation after radiation.

## Discussion

Hematopoietic injury is a common and severe complication of radiation treatment for malignant carcinoma, and can lead to anemia, infection, and hemorrhage. Cytokines serve as one of the most effective drugs for the treatment of hematopoietic dysfunction. However, irradiated hematopoietic cells exhibit a decreased proliferative response toward cytokines. Furthermore, multiple cytokines must be administered to promote the recovery of hematopoiesis, increasing the risk of adverse events and the patients’ financial burden [3,4]. Seeking an efficacious irradiation-resistance agent that promotes hematopoiesis with less severe adverse events could greatly improve the therapeutic efficacy of radiation treatment for malignant carcinoma patients.

Preliminary studies indicated that the peptide isolated from *Buthus martensii* scorpion venom could inhibited the growth of H_22_ tumor. When the venom peptide was administered simultaneously with radiation, the inhibiting effect on H_22_ was enhanced and radiation injury on H_22_-bearing mice could be antagonized by peptide too [9]. The further study showed that SVPs stimulated the secretion of multiple cytokines (IL-1 α, SCF, IL-6, and GM-CSF) in irradiated mice and increased the count of peripheral leucocytes, bone marrow karyocytes, and the number of CFUs formed by isolated bone marrow cells [10,11]. These results suggested that scorpion venom peptides possess the effect of radiation injury mitigation and tumor suppression. At present study we choose M-NFS-60 cells, which were routinely and widely used for modeling hematopoietic events, as the target cells. Our study demonstrated that the isolated peptides SVPII enhanced the proliferation of M-NFS-60 cells, especially after irradiation. The CFU count of bone marrow cells from BALB/C mice was significantly increased after 7, 11, and 14 days of SVPII treatment. This effect was further enhanced when SVP was combined with IL-3.

The reversal of radiation-induced hematopoietic suppression relies on the survival of hematopoietic stem/progenitor cells and reactivated proliferation and differentiation. A variety of cytokines are required during the early-stages of these processes. Alternatively, single and multiple cytokine therapy at advanced-stages of radiation-induced hematopoietic suppression exerted no restorative effect [16]. Hérodin F et al. found that many cytokines, including SCF, FLT-3, TPO, IL-3, and SDF-1 can protect animals from irradiation when administered before the onset of severe damage [16]. Thus, short- and long-term survival after irradiation depends on timely treatment with the appropriate combination of cytokines at optimal concentrations. We observed an enhancing efficacy of SVPII and IL-3 on proliferation in both irradiated and unirradiated M-NFS-60 cells, suggesting that SVPII possesses cytokine-like functions. This combination cytokine therapy not only stimulated cell proliferation, but enabled surviving cells to enter the cell cycle after irradiation. Seven days after irradiation, 35% of cells were arrested in S phase. By contrast, a previous study found that 80% of irradiated cells not treated with IL-3 and stem cell factor failed to enter the cell cycle and a significant fraction became apoptotic[17], indicating that cytokines enhance the recovery of hematopoiesis after irradiation possibly by promoting cell cycle re-entry of HSCs and/or hematopoietic progenitor cells (HPCs). In the current study, the proportion of M-NFS-60 cells at S phase was significantly increased after 24 h of SVPII treatment under serum-free conditions, and the number of cells in S phase was even greater after 96 h treatment. This prolonged SVPII treatment induced more M-NFS-60 cells to enter S phase than IL-3 treatment alone.

Cell cycle arrest and apoptosis are the major mechanisms of radiation-induced bone marrow damage. Damage to DNA activates cell cycle checkpoint proteins and cell cycle arrest at G1 or G2. BAF3 cells resisted X-ray- and cytotoxin-induced injury when the culture media was supplemented with IL-3. Treatment with IL-3 exerted no apparent effect on early-stage DNA damage and repair, but played an essential role in preventing the acceleration of DNA fragmentation at the G2 phase block point [15]. In addition, IL-3 can accelerate G2/M phase arrest and prevent apoptosis of mouse hematopoietic progenitor 32D and human UT7 cell lines in response to etoposide, a type II topoisomerase inhibitor [18]. We found that the proportion of IL-3-treated M-NFS-60 cells arrested at G2/M phase was 65.38%, significantly higher than the 31.71% measured in the control group after irradiation, while the percentage of apoptotic cells (31.95%) was higher than in the control group (15.81%). Gottlieb E et al. [19] observed that IL-3 prevented the apoptosis of DA-1 lymphoma cells at a low irradiation dose. However, p53-dependent DA-1 cell apoptosis occurred at a higher radiation dose even in the presence of IL-3. In our investigation, the relatively high radiation dose used may have overcome the effect of IL-3 so that apoptosis still occurred. However, the number of apoptotic M-NFS-60 cells after SVPII treatment was not significantly different from the irradiated control group. In addition, SVPII had a regulatory effect on cell cycle progression similar to IL-3, significantly increasing the proportion of cells at G2-M phase (to 46.27%) and decreasing the number of cells at S phase. Thus, SVPII has advantages over IL-3 for protecting M-NFS-60 cells in response to a relatively high radiation dose. SVP II may prevent DNA fragmentation and apoptosis at G2 checkpoints after irradiation, although additional studies are necessary to test this possibility.

SVPII promoted the proliferation of IL-3-dependent M-NFS-60 cells, while the combined application of SVPII and IL-3 strengthened the proliferation-promoting effect of either agent alone, suggesting that activation of IL-3R pathways may have contributed to the enhanced proliferation of M-NFS-60 cells. Whether the effects of SVPII and IL-3 were functioned via IL-3Rs was studied by measuring IL-3R expression in M-NFS-60 cells. Both FCM and immunofluorescence results indicated that the expression level of IL-3R was upregulated in M-NFS-60 cells after SVPII treatment. A greater increase in IL-3R expression was measured when M-NFS-60 cells were treated with both SVPII and IL-3, and this enhanced expression was observed under both normal M-CSF and low (25%) M-CSF concentrations. Western blotting also indicated that SVPII significantly upregulated the expression of IL-3R, and exhibited a strengthening effect with IL-3, indicating that the proliferation-enhancing effect of SVPII on M-NFS-60 cells is likely due to IL-3R upregulation. The mutated fibroblast cytokine receptor F36VFGFR1 facilitated the expansion of HSCs in vivo and in vitro, while F36VMpl, a mutant thromboietin receptor, promoted the recovery of myeloid hematopoiesis after irradiation. Other receptors serve as novel regulators of hematopoiesis [20]. Monzen S et al. [21] recently reported that the cytokine receptor genes KIT and IL-3R, as well as genes related to early hematopoiesis and oxidation stress, were all upregulated 7 days after irradiation. Streeter PR et al. [22] indicated that the activation of Flt-3 and G-CSF receptors protected HSCs/HPCs from radiation damage. These studies reveal that cytokine receptors play a vital role in regulating and promoting hematopoiesis after irradiation. The current study demonstrated that IL-3R expression in irradiated M-NFS-60 cells was significantly upregulated 48 h after SVPII treatment. This upregulation was further strengthened by addition of IL-3, indicating that the proliferation promoting effect of SVPII on irradiated cells is closely correlated with upregulation of IL-3R. Hence, IL-3R is a potential therapeutic target for maintaining hematopoietic function following irradiation.

## Conclusion

Radiotherapy for cancer patient may lead to hematopoietic failure. Recombinant cytokine treatment is the traditional therapy for mitigating the inhibitory effect of irradiation on hematopoiesis, but cytokine treatment also causes additional adverse events. Thousands of potential agents that confer radiation-resistance have been investigated. The previous investigation demonstrated the radioprotective efficacy and tumor-inhibiting effect of peptides isolated from the scorpion venom of *Buthus Martti Karsch*. In this paper, we have demonstrated that the proliferation of irradiated M-NFS-60 cells was significantly accelerated by scorpion venom peptide II (SPVII) and induced 10-fold greater overexpression of IL-3R in irradiated M-NFS-60 cells than unirradiated cells. All these effects were further enhanced by co-application of IL-3. Similarly, SPVII increased the number of BM-MNC CFUs and this proliferative effect was greater in the presence of SVPII plus IL-3. SPVII can also alter the cell cycle fractions of M-NFS-60 cells (unirradiated and irradiated). The significance of these results is that SVPII possesses the hematopoietic growth factor (HGF)-like effects on irradiated cells and the effect possibly mediated by upregulation of IL-3R. The cytokines similar functions of SVPII and its mechanisms deserve further study.

## Materials and Methods

### Agents and materials

The peptides SVPII and SVPIII (used only in AlamarBlue™ assay) were isolated from the venom of *Buthus Martti Karsch* as described [11]. Recombinant human macrophage colony stimulating factor (rhM-CSF) and recombinant mouse (rm) IL-3 were purchased from PeproTech Co. AlamarBlue™ was purchased from AbD Serotec (Great Britain), and membrane protein isolation kits were from Bio-Rad. An IL-3R antibody was purchased from Abcam Co. Methyl cellulose for CFU assay was from Sigma-Aldrich Co.

### Cell line

The rhM-CSF-dependent cell line M-NFS-60 (CRL-1838™) was purchased from ATCC Co. (USA).

### Experimental procedures

#### M-NFS-60 cell culture and treatment groups

The M-NFS-60 cell line was cultured in PRMI 1640 culture media supplemented with 10% fetal calf serum (FCS), 100 U/ml penicillin, 100 U/ml streptomycin, 5.958 g/L HEPES, and 62 μg/L rhM-CSF. Cells were maintained at 37°C under a 5% CO_2_ atmosphere. The media was changed every other day. Cells were used for experiments in the exponential growth phase. Unirradiated or ^60^Coγ-irradiated M-NFS-60 cells were treated with PBS (control), SVPII or SVPIII alone, IL-3 alone, or SVP plus IL-3 for various durations.

##### Special cell culture methods

M-NFS-60 cells were cultured in serum-free media supplemented with 62 μg/L rhM-CSF for 24 h (serum-free) or treated with 3 mg/L SVP II (SVP II) or 10 μg/L IL-3 (IL-3). The control cells were cultured 24 h in normal medium (24 h control). After 24 h, the cell cycle was analyzed by FCM. After cultured in serum-free media plus rhM-CSF for 24 h, the cells were cultured in normal midium (mentioned above) for an additional 72 h (serum-free) or treated with SVPII 3 mg/L or IL-3 10 μg/L in the same media. The control cells were cultured 96 h in normal medium (96 h control). After 96 h, the cell cycle was analyzed by FCM. Serum-free medium will lessen the influence factors on the cell cycle progression.

##### After irradiation by ^60^Coγ-ray

M-NFS-60 cells were cultured in PRMI 1640 culture media supplemented with 10% FCS, 100 U/ml penicillin, 100 U/ml streptomycin, 5.958 g/L HEPES, and 15.5 μg/L rhM-CSF (25% of the normal M-CSF dose) for 48 h (irradiated control) or treated with 3 mg/L SVPII or 10 μg/L IL-3 for 48 h. Unirradiated cells were cultured 48 h in the same medium were served as control. After 48 h, the cell cycle was analyzed by FCM.

#### Cell irradiation

M-NFS-60 cells were irradiated by ^60^Coγ-ray at 5 Gy using a Gammacell 3000 Elan installation (Nordion Intern. Inc., Canada). Proliferation and cell cycle progression were then analyzed as described below.

#### Preparation of mouse BM-MNCs

All animal experiments in this study were approved by the Institutional Animal Care and Use Committee of Guangzhou Medical University. The BALB/C mice were euthanized with CO_2_ and the femoral bones removed. The femoral bone cavity was washed with low-sugar DMEM medium to harvest bone marrow cells. The cells in DMEM were then slowly added onto the surface of a lymph cell isolation solution and centrifuged at 2000 rpm for 20 min. The annular white layer consisting of monocytes was collected, washed three times in PBS, and resuspended in DMEM at the optimal concentration for each experiment.

#### AlamarBlue™ cell viability assay

The AlamarBlue assay was used to measure the effect of SVP on the proliferation of non-irradiated and irradiated M-NFS-60 cells cultured in suspension. After irradiation or sham treatment, M-NFS-60 cells were washed three times in PRMI 1640 culture media, and the live cells counted using Trypan Blue vital staining. The cell concentration was adjusted to 5 × 10^4^ cells/mL using PRMI 1640 culture media containing 10% FCS and 62 μg/L rhM-CSF, and aliquoted at 80 μL/well in 96-well plates. After 24 h incubation at 37°C, 10 μL PBS (control), SVP (1, 3, or 4 mg/L), IL-3 (10 μg/L), or SVP + IL-3 was added to each well. Each treatment was performed in triplicate in the same 96-well plate. Following control or drug treatment, 10 μL AlamarBlue™ was added to each well and plates incubated at 37°C for 48 h. Optical density (OD) values were measured and the cell proliferation rate calculated.

#### Colony forming unit (CFU) assay

A methyl cellulose half-solid colony formation method was adopted to measure the number of bone marrow mononuclear cell CFUs under different treatment conditions. Treated BM-MNCs (1 × 10^6^ cells/mL) were added into methyl cellulose half-solid medium composed of DMEM, 0.8% methyl cellulose, 30% FCS, 2 mmol/L L-glutamine, and the recombinant cytokines (10 μg/L IL-3 and 62 μg/L rhM-CSF). The CFU number was counted under a microscope after 7, 11, and 14 days of incubation at 37°C in a 5% CO_2_ atmosphere. A mass consisting of more than 50 cells was defined as 1 CFU.

#### Analysis of the cell cycle using FCM

The M-NFS-60 cells (non-irradiated or irradiated) were treated as described. A 0.5 mL cell suspension (1 − 5 × 10^6^/mL) from each treatment group was combined with 2 ml of cooled 70% ethanol and kept overnight at 4°C, centrifuged at 1000 rpm/min, washed in PBS, and incubated in the dark room at 4°C for 30 min with 50 μL RNAse and 450 μL propidium iodide (PI) staining solution. The proportion of cells in each phase of the cell cycle was then determined by PI staining intensity using FACScalibur flow cytometer (Becton Dickinson).

#### Detection of IL-3R expression

##### Cell immunofluorescence

Cultured M-NFS-60 cells on glass slides were washed twice in PBS, fixed in −20°C pre-cooled 100% methanol for 5 min, dried, and then blocked in 5% BSA solution for 1 h at room temperature or overnight in BSA at 4°C. The blocking solution was removed and anti-IL-3R antibody (Abcam, USA) (1:200) added for 1 h at 37°C or overnight at 4°C. After washing in PBS, an FITC-labeled secondary antibody (F-0382; Sigma) was applied at 1:500 in PBS. Slides were then washed in PBS and sealed in glycerol. The expression of IL-3R was detected by immunofluorescence under a laser scanning confocal microscope (Leica DM1 4000B).

##### FCM analysis of IL-3R expression

Cells were treated as described, harvested, and the concentration of M-NFS-60 cells adjusted to between 5 × 10^**6**^ and 1 × 10^7^ cells/mL in PRMI 1640 culture medium. To this cell suspension were added a monoclonal antibody (5–50 μL) and 50 μL inactivated rabbit serum. Cell suspensions were incubated at 4°C for 30 min, washed in PBS, and centrifuged. The supernatants were removed, and the pellets treated with 50 μL of a FITC-conjugated goat anti-mouse antibody, shaken at 4°C for 30 min, washed twice in PBS, centrifuged, and fixed as described in section 3.7.1.

##### Western blotting

Membrane proteins from the different treatment groups were extracted using a Bio-Rad membrane protein extract kit. Total protein concentrations were measured by the Lowry assay and extracts run on 12% SDS-PAGE gels. Separated proteins were electrotransferred to polyvinyl membranes. Membranes were probed with an IL-3R antibody and visualized using chemiluminescence.

### Statistical analysis

The data are expressed as mean ± SD. SPSS statistical software was used to perform *chi*-square analysis. *P* < 0.05 was considered statistically significant.

## Competing interests

The authors declare that they have no competing interests.

## Authors' contributions

YQ contributed to acquisition of data, performed the statistical analysis and wrote the manuscript. LJ and CW contributed to acquisition of data and performed the statistical analysis. YW performed the statistical analysis and provided reagent. TL contributed to acquisition of data. BX provided reagent. MZ performed the statistical analysis. WD and TK conceived of the study, participated in its design and coordination, helped to draft the manuscript and provided reagent and equipment and helped to draft the manuscript. All authors read and approved the final manuscript.

## References

[B1] Herodin F Drouet M Cytokine-based treatment of accidentally irradiated victims and new approaches Exp Hematol 2005 33 10 1071 1080 10.1016/j.exphem.2005.04.007 16219528

[B2] Katsumori T Yoshino H Hayashi M Takahashi K Kashiwakura I Inductive potential of recombinant human granulocyte colony-stimulating factor to mature neutrophils from x-irradiated human peripheral blood hematopoietic progenitor cells Biol Pharm Bull 2009 32 11 1849 1853 10.1248/bpb.32.1849 19881296

[B3] Danova M Barni S Del Mastro L Danesi R Pappagallo GL Optimal use of recombinant granulocyte colony-stimulating factor with chemotherapy for solid tumors Expert Rev Anticancer Ther 2011 11 8 1303 1313 10.1586/era.11.72 21916584

[B4] Lyman GH Dale DC Long-term outcomes of myeloid growth factor treatment J Natl Compr Canc Netw 2011 9 8 945 952 21900223 10.6004/jnccn.2011.0077

[B5] Singh VK Brown DS Kao TC Tocopherol succinate: a promising radiation countermeasure Int Immunopharmacol 2009 9 12 1423 1430 10.1016/j.intimp.2009.08.020 19735742

[B6] Lee JE Seo I Jeong SJ Koh W Jung JH Kwon TR Lee HJ Han I Lee HJ Lee EO Kim SH Jung HJ Lu J Kim SH Herbal cocktail ka-mi-kae-kyuk-tang stimulates mouse bone marrow stem cell hematopoiesis and janus-activated kinase 2/signal transducer and activator of transcription 5 pathway Am J Chin Med 2011 39 6 1235 1252 10.1142/S0192415X11009524 22083993

[B7] Singh VK Grace MB Parekh VI Whitnall MH Landauer MR Effects of genistein administration on cytokine induction in whole-body gamma irradiated mice Int Immuno pharmacol 2009 9 12 1401 1410 10.1016/j.intimp.2009.08.012 19716438

[B8] Chen T Burke KA Zhan Y Wang X Shibata D Zhao Y IL-12 facilitates both the recovery of endogenous hematopoiesis and the engraftment of stem cells after ionizing radiation Exp Hematol 2007 35 2 203 213 10.1016/j.exphem.2006.10.002 17258069

[B9] Kong TH Wei L Han XF Dong WH Primary observation on the effect of APBMV on tumor weight and general physical condition of hepatoma 22-bearing mice after radiotherapy Chin J Radio Med Prot 2000 20 5 313 316

[B10] Dong WH Wang LN Kong TH He YJ Scorpion venom peptides accelerate hematopoietic recovery of myelosuppression in irradiated mice Am J Chin Med 2009 37 4 701 712 10.1142/S0192415X0900717X 19655408

[B11] He YJ Kong TH Dong WH Scorpion venom polypeptide accelerates irradiated hematopoietic cells proliferation Pathophysiology 2009 16 4 253 258 10.1016/j.pathophys.2009.02.012 19285842

[B12] Nakoinz I Lee MT Weaver JF Ralph P Differentiation of the IL-3-dependent NFS-60 cell line and adaption to growth in macrophage colony-stimulating factor J Immunol 1990 145 3 860 864 2142710

[B13] Wagemaker G Van Gils FC Burger H Dorssers LC Van Leen RW Persoon NL Wielenga JJ Heeney JL Knol E Highly increased production of bone marrow-derived blood cells by administration of homologous interleukin-3 to rhesus monkeys Blood 1990 76 11 2235 2241 2257298

[B14] Robin C Ottersbach K Durand C Peeters M Vanes L Tybulewicz V Dzierzak E An unexpected role for IL-3 in the embryonic development of hematopoietic stem cells Dev Cell 2006 11 2 171 180 10.1016/j.devcel.2006.07.002 16890157

[B15] Collins MK Marvel J Malde P Lopez-Rivas A Interleukin 3 protects murine bone marrow cells from apoptosis induced by DNA damaging agents J Exp Med 1992 176 4 1043 1051 10.1084/jem.176.4.1043 1402650 PMC2119402

[B16] Hérodin F Bourin P Mayol JF Lataillade JJ Drouet M Short-term injection of antiapoptotic cytokine combinations soon after lethal gamma -irradiation promotes survival Blood 2003 101 7 2609 2616 10.1182/blood-2002-06-1634 12468435

[B17] Vávrová J Vokurková D Mareková M Bláha M Jebavý L Filip S Antiapoptotic cytokine IL-3 + SCF + FLT3L influence on proliferation of gamma-irradiated AC133+/CD34+ progenitor cells Folia Biol (Praha) 2002 48 2 51 57 12002675 10.14712/fb2002048020051

[B18] Jin ZH Kurosu T Yamaguchi M Arai A Miura O Hematopoietic cytokines enhance Chk1-dependent G2/M checkpoint activation by etoposide through the Akt/GSK3 pathway to inhibit apoptosis Oncogene 2005 24 12 1973 1981 10.1038/sj.onc.1208408 15674326

[B19] Gottlieb E Lindner S Oren M Relationship of sequence-specific transactivation and p53-regulated apoptosis in interleukin 3-dependent hematopoietic cells Cell Growth Differ 1996 7 3 301 310 8838860

[B20] Weinreich MA Lintmaer I Wang L Liggitt HD Harkey MA Blau CA Growth factor receptors as regulators of hematopoiesis Blood 2006 108 12 3713 3721 10.1182/blood-2006-01-012278 16902155 PMC1895457

[B21] Monzen S Tashiro E Kashiwakura I Megakaryocytopoiesis and thrombopoiesis in hematopoietic stem cells exposed to ionizing radiation Radiat Res 2011 176 6 716 724 10.1667/RR2725.1 22026586

[B22] Streeter PR Dudley LZ Fleming WH Activation of the G-CSF and Flt-3 receptors protects hematopoietic stem cells from lethal irradiation Exp Hematol 2003 31 11 1119 1125 14585378

